# Pembrolizumab plus lenvatinib with or without hepatic arterial infusion chemotherapy in selected populations of patients with treatment-naive unresectable hepatocellular carcinoma exhibiting PD-L1 staining: a multicenter retrospective study

**DOI:** 10.1186/s12885-021-08858-6

**Published:** 2021-10-19

**Authors:** Song Chen, Bo Xu, Zhiqiang Wu, Pengfei Wang, Weiguang Yu, Zhiyong Liu, Xiaoyong Huang, Yanqing Wu, Tengfei Li, Wenbo Guo

**Affiliations:** 1 Department of Invasive Technology, The First Affiliated Hospital, Sun Yat-sen University, No. 58, Zhongshan 2nd Road, Yuexiu District, Guangzhou, 510080 China; 2 Department of Cardiothoracic Surgery, The First Affiliated Hospital, Sun Yat-sen University, No. 58, Zhongshan 2nd Road, Yuexiu District, Guangzhou, 510080 China; 3 Department of Emergency Medicine, The Sun Yat-sen Memorial Hospital of Sun Yat-sen University, No. 107, Yanjiang West Road, Haizhu District, Guangzhou, 510120 China; 4 Department of Orthopedics, The First Affiliated Hospital, Sun Yat-sen University, No. 58, Zhongshan 2nd Road, Yuexiu District, Guangzhou, 510080 China; 5 Department of Oncology, Henan Provincial Tumor Hospital, The Affiliated Cancer Hospital of Zhengzhou University, No. 127, Dongming Road, Jinshui District, Zhengzhou, 450003 China; 6 Department of Hepatic Surgery, Zhongshan Hospital, Fudan University, No. 180, Fenglin Road, Xuhui District, Shanghai, 20032 China; 7 Department of Thyroid Breast Surgery, The First Affiliated Hospital, Sun Yat-sen University, No. 58, Zhongshan 2nd Road, Yuexiu District, Guangzhou, 510080 China; 8 Department of Cardiology, The First Affiliated Hospital, Sun Yat-sen University, No. 58, Zhongshan 2nd Road, Yuexiu District, Guangzhou, 510080 China

**Keywords:** Pembrolizumab, Lenvatinib, Hepatic arterial infusion chemotherapy, Hepatocellular carcinoma, Survival

## Abstract

**Background:**

Not all patients with unresectable hepatocellular carcinoma (uHCC) benefit from treatment with immune checkpoint inhibitors and molecular-targeted agents. The aim of this retrospective study was to assess the efficacy and safety of pembrolizumab plus lenvatinib plus hepatic arterial infusion chemotherapy (HAIC) versus pembrolizumab plus lenvatinib in selected populations of patients with treatment-naive uHCC exhibiting programmed cell death ligand-1 (PD-L1) staining.

**Methods:**

Consecutive patients with treatment-naive uHCC exhibiting PD-L1 staining who were treated with pembrolizumab plus lenvatinib plus HAIC (PLH) or pembrolizumab plus lenvatinib (PL) were retrospectively identified from our medical centres from 2018 to 2021. HAIC involved oxaliplatin, fluorouracil, and leucovorin (FOLFOX). Follow-up occurred every 3 weeks for 1 year and then every 6 weeks thereafter. The primary endpoints included overall survival (OS) and progression-free survival (PFS). Secondary endpoints were the frequency of key adverse events (AEs).

**Results:**

In total, 248 treatment-naive patients were retrospectively reviewed, 78 of whom were ineligible on the basis of the current criteria. Thus, 170 patients (PLH: *n* = 84, median age 52 years [range, 42–67]; PL: *n* = 86, 53 years [range, 43–69]) were eligible for the analysis. The median follow-up was 18.6 months (range, 1–26). At the final follow-up, the median OS was 17.7 months (95% confidence interval [CI], 15.2–18.3) in the PLH group versus 12.6 months (95% CI, 11.1–13.7) in the PL group (hazard ratio [HR] 0.52; 95% CI, 0.36–0.75; *p* = 0.001). A significant difference was also detected in the median PFS (10.9 months [95% CI, 8.7–11.4] for PLH vs. 6.8 months (95% CI, 5.2–7.4) for PL; HR 0.61, 95% CI, 0.43–0.85; *p* = 0.001). Significant differences in the rate of the key AEs were noted between groups (79.8% for PLH vs. 62.8% for PL, *p* = 0.015), but these AEs were controllable.

**Conclusions:**

Among selected populations of patients with treatment-naive uHCC exhibiting PD-L1 staining, the PLH regimen may substantially improve the survival benefits compared with the PL regimen with a controllable safety profile.

## Background

Hepatocellular carcinoma (HCC) accounts for approximately 90% of all primary liver cancers and is the 4th leading cause of cancer-related death [1]. In China, HCC is primarily attributed to chronic inflammation resulting from hepatitis B virus infection [2]. Most patients are newly diagnosed with unresectable disease. Thus, treatment options are limited, and the prognosis is poor [2, 3]. The management of uHCC with inherent “immune escape” remains controversial [3, 4]. Several regimens [5–8] have been revealed to improve the survival of patients with uHCC. However, based on previously routine radiotherapy and/or chemotherapy, the majority of these patients exhibit a poor prognosis and ultimately die from uHCC within 1–2 years, resulting in a push for the development of other feasible therapies [9, 10].

uHCC tends to be characterized by intrinsic immunosuppression and overexpression of immune checkpoints, which mainly involve the programmed death 1 (PD-1) pathway and the cytotoxic T-lymphocyte-associated protein 4 (CTLA-4) pathway [11, 12]. Although the management of uHCC is precipitously evolving with numerous new therapeutic options, immunotherapy still plays a key role even in combination regimens [13–15]. Importantly, PD-1 pathway blockade with immune checkpoint inhibitors has emerged as a promising option to potentially delay the progression of tumours and improve survival benefit [16, 17]. Pembrolizumab, a highly selective IgG4-kappa humanized monoclonal antibody against the PD-1 receptor, can release the antitumour activity of pre-existing tumour-specific T-cell immunity and has been approved as a second-line agent for uHCC patients who cannot tolerate or experience disease progression during or following treatment with drugs targeting the Raf/Mek/Erk pathway on the basis of the Keynote-224 trial [12]. The immunomodulatory effect of lenvatinib (a receptor tyrosine kinase inhibitor) on tumour microenvironments might contribute to antitumour activity when combined with PD-1 signalling inhibitors in HCC [18, 19]. Combining pembrolizumab with a lenvatinib regimen for uHCC has been reported in a multicentre, open-label trial [16] of 104 patients with uHCC that assessed the clinical outcomes of pembrolizumab plus lenvatinib. In this trial, the median overall survival (OS) and median progression-free survival (PFS) were 22 months and 8.6 months, respectively. Grade ≥ 5 adverse events (AEs) occurred in 3% of patients. Their conclusion revealed that the combination has promising antitumour activity in uHCC with an anticipated safety profile.

Although pembrolizumab plus lenvatinib has been demonstrated to improve survival benefits for patients with uHCC, the regimen of pembrolizumab plus lenvatinib in combination with hepatic arterial infusion chemotherapy (HAIC) is not commonly employed in uHCC. Whether the incorporation of HAIC into pembrolizumab plus lenvatinib results in improved survival in patients with uHCC remains an open question to date; however, the efficacy of HAIC has been the focus of debate due to high response rates and encouraging survival rates for uHCC patients [20]. Furthermore, given that pembrolizumab represents diverse anticancer activities for a subset of uHCC [21], the need to stratify patients based on PD-1 expression status and the actual antitumour efficacy of pembrolizumab plus lenvatinib plus HAIC, even in a biomarker-selected HCC setting, has not been evaluated. In this study, we performed a multicentre retrospective study to assess the efficacy and safety of pembrolizumab plus lenvatinib plus HAIC versus pembrolizumab plus lenvatinib in selected populations of patients with treatment-naive uHCC exhibiting programmed death ligand 1 (PD-L1) staining.

## Methods

### Data

Consecutive patients with treatment-naive uHCC whose tumours expressed PD-L1 and who initially underwent the pembrolizumab plus lenvatinib plus HAIC (PLH) or pembrolizumab plus lenvatinib (PL) regimen from March 1, 2018, to March 31, 2021, for whom baseline data were available, were retrospectively identified from the First Affiliated Hospital, Sun Yat-sen University, the Sun Yat-sen Memorial Hospital of Sun Yat-san University, Henan Provincial Tumor Hospital, the Affiliated Cancer Hospital of Zhengzhou University and Zhongshan Hospital, Fudan University. A review of the patient’s medical records was executed independently by the four authors (B X, Pf W, Zy Land Xy H) to extract endpoint data. Inclusion criteria were as follows: imaging and/or histologically confirmed HCC; an HCC classification of Barcelona Clinic Liver Cancer (BCLC) B or C; unresectable evaluated by two or more experienced surgical specialists; Child-Pugh A or B; PD-L1 expression staining (combined positive score [CPS] ≥1) as determined by an FDA-approved test; PD-L1 expression was assessed using the PD-L1 IHC 22C3 pharmDx assay (Agilent Technologies) [16]; Eastern Cooperative Oncology Group (ECOG) performance status of 0 or 1; at least one measurable target lesion by the modified Response Evaluation Criteria in Solid Tumours (mRECIST); anti-HBV therapy was required; and appropriate bone marrow function. Major exclusion criteria involved absent baseline data; previous agent(s) targeting T-cell costimulation or checkpoint pathways; no evaluable CPS; previous tumour-related interventions (such as hormonal therapy, chemotherapy, radiofrequency ablation, radiotherapy, microwave coagulation therapy, or surgery); discontinuation of PLH or LH, irrespective of drug-related AEs; cachexia; severe medical system diseases (such as systemic inflammatory response syndrome, hepatic encephalopathy, stubborn anaemia, or abnormal blood coagulation); surgical emergency (such as intestinal obstruction, intestinal perforation, or gastrointestinal haemorrhage); multiple organ dysfunction syndrome; other malignant tumours; and psychosis.

### Study design and treatment

A retrospective multicentre study was conducted in which eligible patients had received either the PLH or PL regimen for the treatment of uHCC exhibiting PD-L1 expression staining. The decision to manage using PLH or PL was made by medical experts on a case-by-case basis. In the PLH group, patients were administered 200 mg pembrolizumab intravenously once every 3 weeks [12] and 8–12 mg lenvatinib once daily orally [22] followed by HAIC every 3 weeks. HAIC consisted of 85 mg/m^2^ oxaliplatin from hour 0 to 2 on day 1; 400 mg/m^2^ fluorouracil bolus at hour 3 and 2400 mg/m^2^ fluorouracil over 46 h on days 1 and 2; and 400 mg/m^2^ leucovorin from hour 2 to 3 on day 1 as reported by He et al. [8]. In the PL group, patients were administered 200 mg pembrolizumab intravenously once every 3 weeks and 8–12 mg lenvatinib once daily orally. Treatment was continued until disease progression, intolerable toxicity, withdrawal, or death.

### Outcomes and assessments

The primary endpoints were OS and PFS. OS was defined as the time of initiation of medication to death from any cause, and PFS, defined as the time of initiation of medication to disease progression or death from any cause, whichever came first. Tumour response was assessed in accordance with RECIST version 1.1 and mRECIST per independent imaging review. Analysis of patterns of response was performed based on computed tomography (CT) or magnetic resonance imaging (MRI). The diagnosis of extrahepatic metastasis was primarily based on CT or MRI. In some cases, the diagnosis of extrahepatic metastasis was based on positron emission tomography-CT (PET-CT). Secondary endpoints were the rate of major AEs that were graded according to the National Cancer Institute Common Terminology Criteria for Adverse Events, version 4.03. The major AE evaluations were performed continuously throughout the follow-up period. Information regarding these AEs was collected from the time of initiation of PLH or PL therapy. PD-L1 expression was analysed using immunohistochemistry. The measurement of tumour PD-L1 expression staining was consistent with a previous description [11]. Follow-up was performed every 3 weeks for 1 year and then every 6 weeks thereafter to monitor endpoint parameters until disease progression, intolerable AEs, or death. CT or MRI was performed every 6 weeks. Additional follow-up was permitted when clinically indicated.

### Statistical analysis

Normally distributed continuous variables were compared by t-test, and abnormally distributed continuous variables were compared using the Mann-Whitney U test. Categorical variables were compared by Pearson’s χ^2^ test or Fisher’s exact test. Survival was estimated using the Kaplan-Meier method with a log-rank test. All variables with *p* < 0.05 in univariate analyses were incorporated into multivariate analyses. Univariate and multivariate analyses were performed using the Cox proportional hazards model. Here, age, ECOG performance status, HCC aetiology, Child-Pugh A or B, Albumin–Bilirubin (ALBI) grade (1/> 1), liver cirrhosis, PD-L1 expression, tumour thrombus, extrahepatic metastasis, and number of metastatic sites were used as covariates, and intervention served as the time-dependent factor. The median follow-up was calculated using the reverse Kaplan-Meier method. A two-tailed *p*-value of < 0.05 was used as a threshold for significance. Statistical analyses were performed primarily using SPSS 26.0 (IBM, Inc., NY). The survival curves for both groups were outlined using GraphPad Prism 8.0 (La Jolla, California, USA).

## Results

### Demographic characteristics

**Fig. 1:**
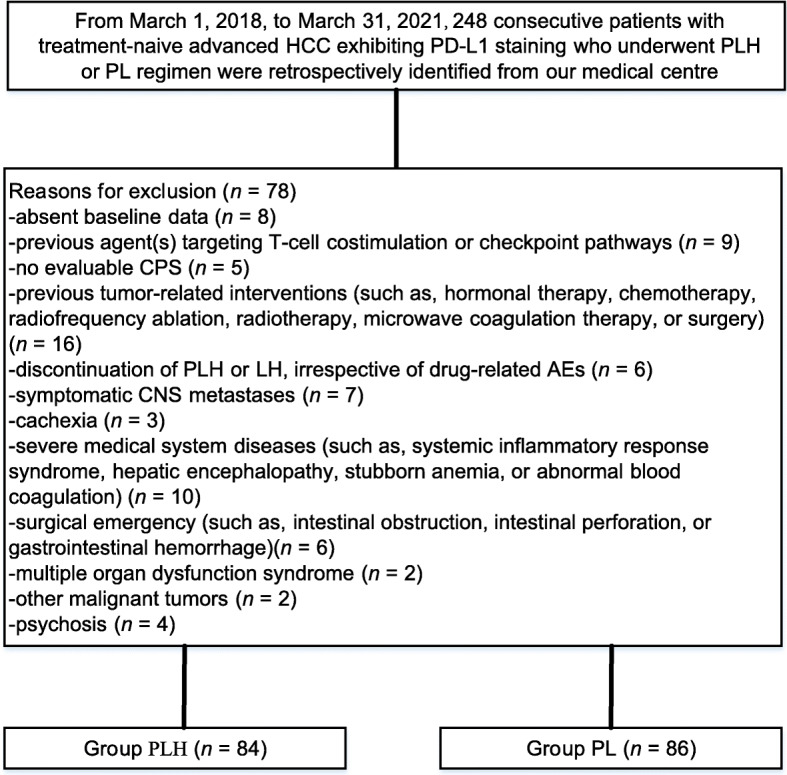
Flow diagram exhibiting the methods applied to identify objects to assess the efficacy and safety of pembrolizumab plus lenvatinib plus hepatic arterial infusion chemotherapy (HAIC) versus pembrolizumab plus lenvatinib in selected populations of patients with treatment-naive uHCC exhibiting programmed cell death ligand-1 (PD-L1) staining

We identified 248 consecutive patients with treatment-naive uHCC exhibiting PD-L1 staining who underwent the PLH or PL regimen, of whom 78 were excluded based on the current criteria. Finally, a total of 170 patients who met the criteria were included in the study, of whom 84 received the PLH regimen and 86 received the PL regimen, as presented in Fig. 1. Table 1 summarizes the patient’s clinical characteristics. The median age was 52 years (range, 42–67) in the PLH group and 53 years (range, 43–69) in the PL group. BCLC status was B in 26.2% and C in 73.8% of patients receiving PLH versus B in 24.4% and C in 75.6% of patients receiving PL (*p* = 0.791). The PD-L1 CPS was 1–20 in 50.0%, 20–50 in 32.1%, and 50–100 in 17.9% of patients receiving PLH versus 1–20 in 52.3%, 20–50 in 27.9%, and 50–100 in 19.8% of patients receiving PL (*p* = 0.906). Absent tumour thrombus, branch of portal vein thrombus, and main portal vein thrombus were 41.7%, 38.142.8%, and 36.9%15.5% in the PLH group versus 36.0%, 43.051.2%, and 33.7%12.8% in the PL group, respectively (*p* = 0.695). The two cohorts were well balanced in the present study. The duration of drugs was 15 months (range, 1–33) in the PLH group and 12 months (range, 2–33) in the PL group. The median number of treatment cycles was 25 (range, 1–36) for patients undergoing PLH and 17 (range, 1–37) for those who underwent PL.

**Table 1:** Baseline data of patients who were treated with PLH or PL regimen

Variable	PLH (*n* = 84)	PL (*n* = 86)	*p*-value
Age, years			
Median (range)	52 (42–67)	53 (43–69)	0.124^*a*^
Sex, n (%)			0.575^*b*^
Male	72 (85.7)	4771 (82.6)	
Female	12 (14.3)	15 (17.4)	
BMI, kg/m^2^			
Median (range)	25.2 (16.7–42.1)	25.3 (16.2–41.8)	0.309^*a*^
ECOG performance status, n (%)			0.551^*b*^
0*	38 (45.2)	35 (40.7)	
1**	46 (54.8)	51 (59.3)	
Time since diagnosis, month(s)	6 (1–12)	6 (1–14)	0.435^*a*^
HCC aetiology, n (%)			0.556^*b*^
Hepatitis B virus	45 (53.6)	48 (55.8)	
Hepatitis C virus	22 (26.2)	26 (30.2)	
Without viral hepatitis	17 (20.2)	12 (14.0)	
AFP (ng/ml)	3984.0 (82.0–49,534.0)	4022.0 (79.0–51,462.0)	0.101^*a*^
Child-Pugh, n (%)			0.616^*b*^
A	71 (84.5)	75 (87.2)	
B	13 (15.5)	11 (12.8)	
BCLC			0.791^*b*^
B	22 (26.2)	21 (24.4)	
C	62 (73.8)	65 (75.6)	
ALBI grade, n (%)			0.464^*b*^
1	20 (23.8)	22 (25.6)	
2	56 (66.7)	60 (69.8)	
3	8 (9.5)	4 (4.6)	
Liver cirrhosis, n (%)			0.954^*b*^
Absent	27 (32.1)	28 (32.6)	
Present	57 (67.9)	58 (67.4)	
PD-L1 expression (CPS cut-off values)^#^, n (%)			0.906^*b*^
1–20	42 (50.0)	45 (52.3)	
20–50	27 (32.1)	24 (27.9)	
50–100	15 (17.9)	17 (19.8)	
Tumour thrombus, n (%)			0.695^*b*^
Absent	35 (41.7)	31 (36.0)	
Branch of portal vein	36 (42.8)	44 (51.2)	
Main portal vein	13 (15.5)	11 (12.8)	
Extrahepatic metastasis, n (%)			0.543^*b*^
Absent	64 (76.2)	62 (72.1)	
Present	20 (23.8)	24 (27.9)	
Duration of drugs (months)	15 (1–33)	12 (2–33)	0.081^*a*^
Number of metastatic sites, n (%)			0.969^*b*^
< 3	7 (8.3)	6 (7.0)	
≥ 3	64 (76.2)	68 (79.1)	
Unclear	13 (15.5)	12 (13.9)	

^a^Independent samples t-test; ^b^Mann-Whitney U test;

0*, Fully active, able to carry on all pre-disease performance without restriction; 1**, Restricted in physically strenuous activity but ambulatory and able to carry out work of a light or sedentary nature; ^#^patients with high tumor programmed cell death ligand-1 expression had a better outcome

*PLH* pembrolizumab plus lenvatinib plus hepatic arterial infusion chemotherapy; *PL* pembrolizumab plus lenvatinib; *BMI* body mass index; *ECOG* Eastern Collaborative Oncology Group; *HCC* hepatocellular carcinoma; *AFP* alpha fetoprotein; *BCLC* Barcelona clinic liver cancer; *ALBI* albumin–bilirubin; *PD-L1* programmed cell death ligand-1; *CPS* combined positive score

### Efficacy

**Fig. 2:**
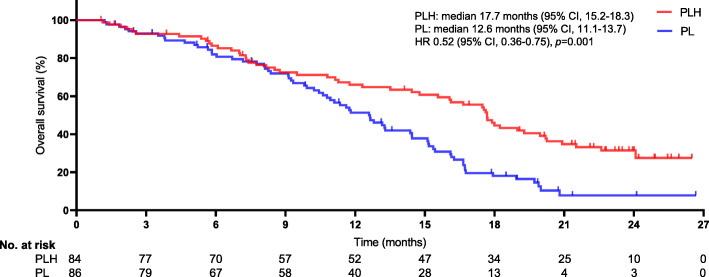
Kaplan-Meier curves for overall survival. The median overall survival was 17.7 months (95% confidence interval [CI], 15.2–18.3) for PLH and 12.6 months (95% CI, 11.1–13.7) for PL (HR 0.52, 95%CI, 0.36–0.75; *p* = 0.001). *The hazard ratio was calculated using a Cox proportional hazards model, with the age, ECOG performance status, time since diagnosis, HCC aetiology, Child-Pugh A or B, Liver cirrhosis, PD-L1 expression, tumor thrombus, central nervous system metastasis, and number of metastatic sites used as covariates and therapy as the time-dependent factor

**Fig. 3:**
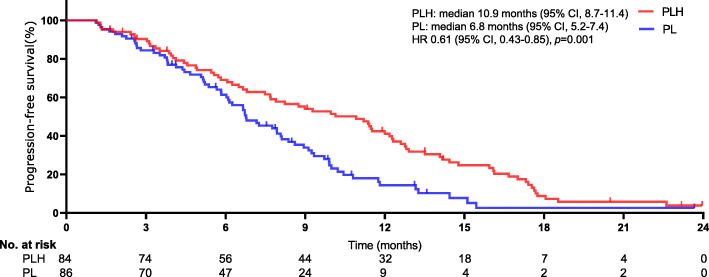
Kaplan-Meier curves for progression-free survival. The median progression-free survival was 10.9 months (95% confidence interval [CI], 8.7–11.4) for PLH and 6.8 months (95% CI, 5.2–7.4) for PL (HR 0.61, 95%CI, 0.43–0.85; *p* = 0.001). *The hazard ratio was calculated using a Cox proportional hazards model, with the age, ECOG performance status, time since diagnosis, HCC aetiology, Child-Pugh A or B, Liver cirrhosis, PD-L1 expression, tumor thrombus, central nervous system metastasis, and number of metastatic sites used as covariates and therapy as the time-dependent factor

The median follow-up was 18.6 months (range, 1–26). Tumour response in patients experiencing PLH or PL were showed in Table 2. Between-group remarkable distinctions were detected in objective response rate (ORR) (all *p* < 0.05). In the PLH group, ORR was 46.4% (95% CI, 29.3–54.6%) by RECIST version 1.1 and 59.5% (95% CI, 42.7–58.7%) by mRECIST. In the PL group, ORR was 30.2% (95% CI, 22.5–44.7%) by RECIST version 1.1 and 41.9% (95% CI, 33.7–57.1%) by mRECIST. At the final follow-up, 121 (71.2%) deaths occurred (53 [63.1%] PLH-treated patients vs. 68 [79.1%] PL-treated patients). The 3-, 6-, and 12-month OS rates were 92.7, 86.5, and 64.7%, respectively, in PLH-treated patients and 92.9, 81.9, and 51.3%, respectively, in PL-treated patients. A borderline significant distinction was observed in the median OS between the two cohorts (17.7 months [95% CI, 15.2–18.3] in the PLH-treated cohort vs. 12.6 months [95% CI, 11.1–13.7] in the PL-treated cohort), as shown in Fig. 2. PLH had a remarkable improvement in the median OS compared with PL, and PLH might be associated with a significant 48% lower risk of death than PLH (HR 0.52, 95% CI, 0.36–0.75; *p* = 0.001). A noteworthy difference of 5.1 months in the median OS was noted, and the superiority of PLH over PL tended to be positive, as the separation of the two curves continued until the final follow-up. Moreover, a distinct dissimilarity in the median PFS between the two cohorts was observed (10.9 months [95% CI, 8.7–11.4] in the PLH-treated cohort vs. 6.8 months [95% CI, 5.2–7.4] in the PL-treated cohort [HR 0.61, 95% CI, 0.43–0.85; *p* = 0.001]), as shown in Fig. 3. Of the 170 patients, treatment interruption occurred in 23 (13.5%) patients mainly due to tumour progression (17 of 84 patients in the PLH-treated cohort vs. 6 of 86 patients in the PL-treated cohort, *p* = 0.026). Although more cases suffered tumour progression in the PLH-treated cohort, a remarkable delay was observed in the time interval for tumours to progress in this cohort, which may be associated with the significantly longer PFS.

**Table 2:** Tumour response in patients experiencing PLH or PL

	PLH (*n* = 84)		PL (*n* = 86)		*p-value*^*c*^	*p-value*^***d***^
	RECIST version 1.1	mRECIST	RECIST version 1.1	mRECIST		
ORR^**a**^, n (%)	39 (46.4)	50 (59.5)	26 (30.2)	36 (41.9)	0.030	0.022
95% CI	29.3–54.6	42.7–58.7	22.5–44.7	33.7–57.1		
Overall response^**b**^, n (%)						
CR	5 (6.0)	13 (15.5)	1 (1.2)	8 (9.3)	0.092	0.223
PR	34 (40.5)	37 (44.0)	25 (29.1)	28 (32.6)	0.119	0.124
SD	37 (44.0)	24 (28.6)	46 (53.5)	35 (40.7)	0.220	0.098
PD	7 (8.3)	9 (10.7)	11 (12.7)	12 (13.9)	0.346	0.522
NA	1 (1.2)	1 (1.2)	3 (3.5)	3 (3.5)	0.319	0.319

NOTE. RECIST version 1.1 or mRECIST was assessed per independent imaging review

^a^defined as the proportion of individuals who had a confirmed CR or PR per independent imaging review; ^b^involved evaluation of the change in tumour burden inside and outside the liver; ^c^the comparison between the two groups is based on the RECIST version 1.1; ^d^ the comparison between the two groups is based on the mRECIST

*PLH* pembrolizumab plus lenvatinib plus hepatic arterial infusion chemotherapy; *PL* pembrolizumab plus lenvatinib; *RECIST version 1.1* Response Evaluation Criteria in Solid Tumours version 1.1; *mRECIST* modified RECIST; *ORR* objective response rate; *CI* confidence interval; *CR* complete response; *PR* partial response; *SD* stable disease; *PD* progressive disease; *NA* not assessable

**Fig. 4:**
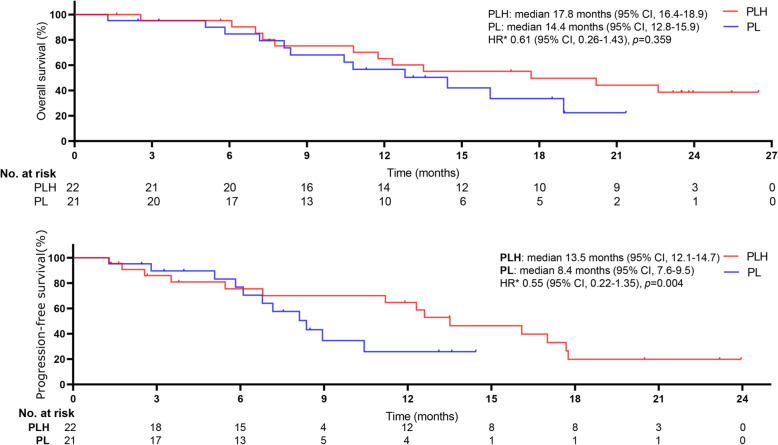
Kaplan-Meier curves for subgroup analysis in selected populations of treatment-naive advanced HCC patients with Barcelona Clinic Liver Cancer B between groups

**Fig. 5:**
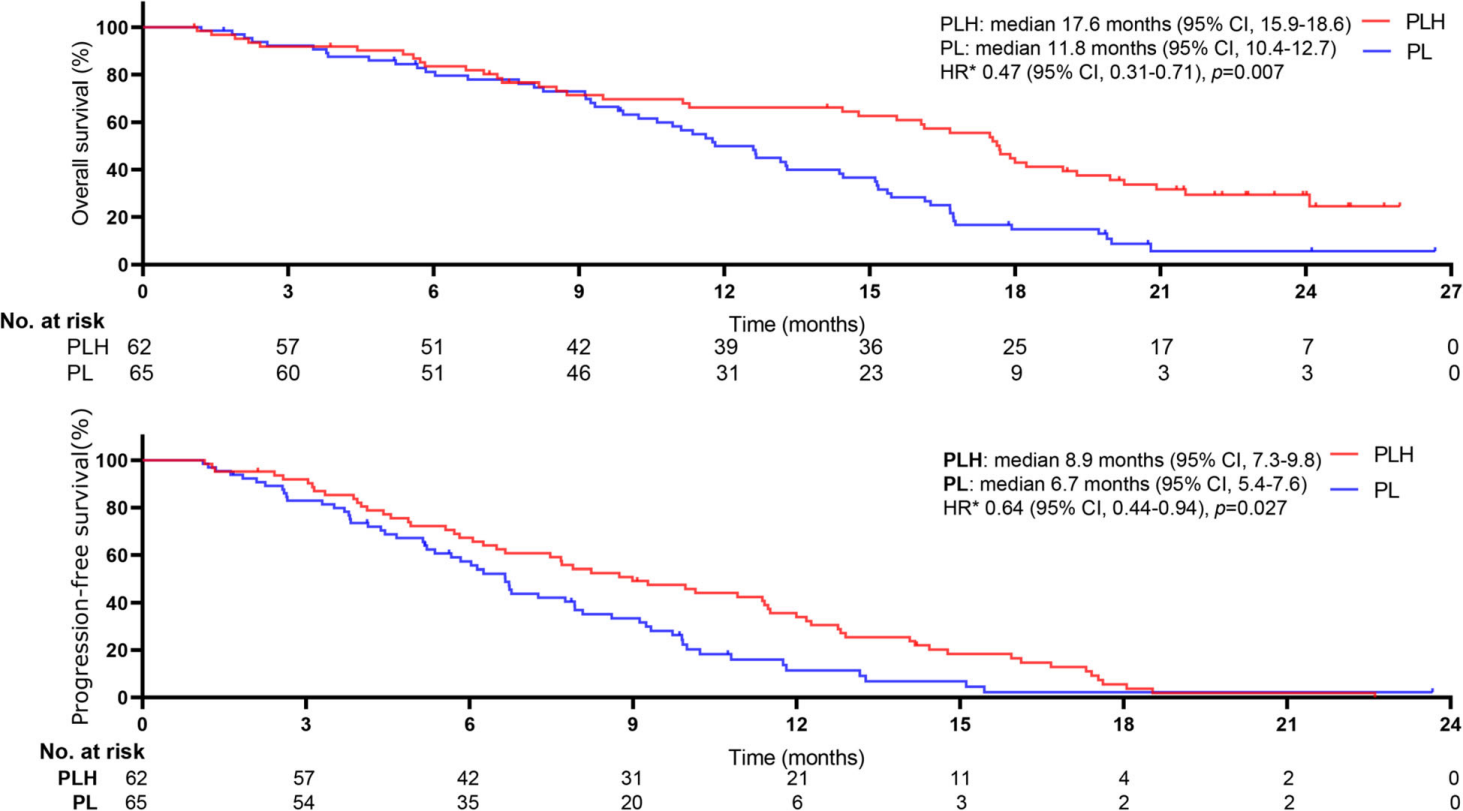
Kaplan-Meier curves for subgroup analysis in selected populations of treatment-naive advanced HCC patients with Barcelona Clinic Liver Cancer C between groups

For patients with BCLC (B), median OS was 17.8 months (95% CI, 16.4–18.9) for PLH and 14.4 months (95% CI, 12.8–15.9) for PL (HR 0.61, 95%CI, 0.26–1.43; *p* = 0.359); median PFS was 13.5 months (95%CI, 12.1–14.7) for PLH and 8.4 months (95% CI, 7.6–9.5) for PL (HR 0.55, 95%CI, 0.22–1.35; *p* = 0.004) (Fig. 4). For patients with BCLC (C), median OS was 17.6 months (95% CI, 15.9–18.6) for PLH and 11.8 months (95% CI, 10.4–12.7) for PL (HR 0.47, 95%CI, 0.31–0.71; *p* = 0.007); median PFS was 8.9 months (95% CI, 7.3–9.8) for PLH and 6.7 months (95% CI, 5.4–7.6) for PL (HR 0.64, 95%CI, 0.44–0.94; *p* = 0.027) (Fig. 5).

We performed a subgroup comparison based on the CPS scores. For patients with PD-L1 CPS ≥50, the median OS was 18.1 months in the PLH-treated cohort compared with 13.3 months in the PL-treated cohort (HR 0.75; *p* = 0.003). For patients with PD-L1 CPS ≥20, the median OS was 17.5 months in the PLH-treated cohort compared with 12.2 months in the PL-treated cohort (HR 0.26; *p* = 0.002). For patients with PD-L1 CPS ≥1, the median OS was 16.7 months in the PLH-treated cohort compared with 11.7 months in the PL-treated cohort (HR 0.48, *p* = 0.001). The higher the CPS implying the occurrence of a strong immune response to pembrolizumab, the more likely the patients will benefit from pembrolizumab.

### Univariate and multivariate analysis for survival

Tables 3 and 4 demonstrate the univariate and multivariate analyses for survival. Multivariate analysis demonstrated that the therapeutic regimen was an independent risk factor for both OS (HR 3.649; 95% CI, 1.592–6.074; *p* = 0.001) and PFS (HR 2.315; 95% CI, 1.211–3.629; *p* = 0.001). Multivariate analysis showed that Child-Pugh (B), and number of metastatic sites (≥3) were risk factors for OS and that AFP ≥ 400 ng/ml was a risk factor for PFS.

**Table 3:** Univariate and multivariate analysis of risk factors for overall survival

Variables	Overall survival					
	Univariate analysis				Multivariate analysis	
	HR	95%CI	*p*	HR	95%CI	*p*
Age (years), </≥60	1.185	1.305–2.129	0.747			
Sex, (male/female)	0.583	0.392–2.470	0.274			
BMI, (kg/m^2^), </≥25	1.191	0.726–1.103	0.125			
ECOG (0/1)	1.182	0.943–2.734	0.282			
Hepatitis B, (yes/no)	1.688	0.769–2.650	0.511			
Hepatitis C, (yes/no)	1.352	0.144–2.013	0.206			
AFP (ng/ml), </≥400	1.566	0.072–2.317	0.114			
Child-Pugh (A/B)	2.403	2.500–8.434	0.005	2.633	1.391–9.648	0.001
BCLC (B/C)	1.390	0.651–2.591	0.171			
Liver cirrhosis (yes/no)	1.772	0.785–3.647	0.663			
PD-L1 expression	1.473	0.860–3.429	0.052			
Tumor thrombus						
Absent						
Branch of portal vein	1.583	0.303–4.752	0.545			
Main portal vein	1.276	0.295–6.298	0.901			
Extrahepatic metastasis (yes/no)	1.292	0.747–3.842	0.118			
Number of metastatic sites (< 3/≥3)	2.104	1.360–4.476	0.007	2.193	1.187–4.366	0.002
Treatment (PLH/PL)	2.499	1.726–4.902	0.005	3.649	1.592–6.074	0.001

*PLH* pembrolizumab plus lenvatinib plus hepatic arterial infusion chemotherapy; *PL* pembrolizumab plus lenvatinib; *BMI* body mass index; *ECOG* Eastern Collaborative Oncology Group; *AFP* alpha fetoprotein; *BCLC* Barcelona clinic liver cancer; P*D-L1* programmed cell death ligand-1

**Table 4:** Univariate and multivariate analysis of risk factors for progression-free survival

Variables	Progression-free survival					
	Univariate analysis			Multivariate analysis		
	HR	95%CI	*p*	HR	95%CI	*p*
Age (years), </≥60	0.606	0.195–1.573	0.336			
Sex, (male/female)	2.440	0.657–3.235	0.253			
BMI, (kg/m^2^), </≥25	1.655	0.878–4.917	0.195			
ECOG (0/1)	1.638	0.266–2.804	0.416			
Hepatitis B, (yes/no)	2.434	0.632–4.161	0.685			
Hepatitis C, (yes/no)	1.841	0.818–3.246	0.287			
AFP (ng/ml), </≥400	1.565	1.124–3.027	0.011	2.917	1.425–4.760	0.002
Child-Pugh(A/B)	2.511	0.944–6.070	0.665			
BCLC (yes/no)	2.628	0.606–3.649	0.705			
Liver cirrhosis (yes/no)	1.298	0.428–2.822	0.691			
PD-L1 expression	1.136	0.738–2.651	0.173			
Tumor thrombus						
Absent						
Branch of portal vein	1.721	0.167–2.810	0.554			
Main portal vein	2.591	0.182–1.321	0.277			
Extrahepatic metastasis (yes/no)	1.782	0.441–3.647	0.287			
Number of metastatic sites (< 3/≥3)	2.368	0.375–3.458	0.748			
Treatment (PLH/PL)	1.779	1.281–2.680	0.002	2.315	1.211–3.629	0.001

*PLH* pembrolizumab plus lenvatinib plus hepatic arterial infusion chemotherapy; *PL* pembrolizumab plus lenvatinib; *BMI* body mass index; *ECOG* Eastern Collaborative Oncology Group; *AFP* alpha fetoprotein; *BCLC* Barcelona clinic liver cancer; *PD-L1* programmed cell death ligand-1

### Safety

During the entire follow-up, the frequency of the key AEs is presented in Table 5. At the final follow-up, no significant differences in the rate of each AE were noted between groups. Treatment-related serious AEs (≥ grade 3) occurred in 6 patients (4 in the PLH versus 2 in the PL group, *p* = 0.391). No treatment-related deaths occurred in either cohort. The most common treatment-related AEs included alanine aminotransferase or aspartate aminotransferase elevation, total bilirubin elevation, decreased platelet, hypertension, fever, fatigue, decreased albumin, leukocytopenia, pain, nausea, and diarrhea, which were mostly grade 1/2. In the PLH group, the frequency of the key AEs of all grades was 79.8% (137 AEs in 67 patients). In the PL group, the frequency of the key AEs of all grades was 62.8% (104 AEs in 54 patients). A higher rate of key AEs was detected in the PLH group than in the PL group (79.8% vs. 62.8%, *p* = 0.015), but these AEs were controllable.

**Table 5:** Treatment-related adverse events

Adverse events	PLH (*n* = 84)	PL (*n* = 86)	HR (95%)	*p-*value
Treatment-related AEs, n (%)				
Hypertension	26 (30.9)	25 (29.1)	1.00 (0.42–1.79)	0.789
Fever	20 (23.8)	15 (17.4)	3.00 (0.29–1.45)	0.306
Fatigue	16 (19.0)	12 (14.0)	2.00 (0.44–3.79)	0.372
Decreased appetite	14 (3.6)	9 (10.5)	1.00 (0.26–2.57)	0.239
Pain	12 (14.3)	10 (11.6)	3.00 (0.28–5.14)	0.607
Nausea	10 (11.9)	8 (9.3)	1.00 (0.11–2.02)	0.583
Diarrhea	10 (11.9)	6 (7.0)	2.00 (0.43–4.22)	0.273
Cough	7 (8.3)	4 (4.7)	1.00 (0.59–2.63)	0.331
Rash	9 (10.7)	6 (7.0)	4.00 (0.88–6.57)	0.392
Pruritus	5 (6.0)	3 (3.5)	1.00 (0.63–2.46)	0.449
Hyperthyroidism	1 (1.2)	1 (1.2)	2.00 (0.47–4.02)	0.987
Hypothyroidism	3 (3.6)	3 (3.5)	2.00 (0.91–3.85)	0.977
Edema peripheral	4 (4.8)	2 (2.3)	1.00 (0.16–2.35)	0.391
Laboratory-related AEs, n (%)				
Leukocytopenia	12 (14.3)	18 (20.9)	2.00 (0.59–2.63)	0.257
Decreased PLT	21 (25.0)	24 (27.9)	1.00 (0.32–3.27)	0.668
ALT or AST elevation	47 (55.9)	52 (60.5)	2.00 (0.71–1.65)	0.552
TBIL elevation	24 (28.6)	26 (30.2)	1.00 (0.37–2.28)	0.813
Decreased ALB	16 (19.0)	19 (22.1)	1.00 (0.45–1.69)	0.624

*PLH* pembrolizumab plus lenvatinib plus hepatic arterial infusion chemotherapy; *PL* pembrolizumab plus lenvatinib; *PLT* platelet; *ALT* alanine aminotransferase; *AST* aspartate aminotransferase; *TBIL* total bilirubin; *ALB* albumin

## Discussion

The findings from the present retrospective study showed that the incorporation of HAIC into the pembrolizumab plus lenvatinib regimen may result in markedly longer PFS for selected populations of patients with treatment-naive uHCC exhibiting PD-L1 staining and significantly longer OS than the pembrolizumab plus lenvatinib regimen with a controllable safety profile. The survival curves among these patients included at an early survival benefit for patients treated using pembrolizumab plus lenvatinib plus HAIC that continued until the final follow-up with a noteworthy 5.1-month difference in median OS.

The findings of the present study are broadly consistent with a retrospective study [2] of 70 patients with advanced HCC that assessed the antitumour efficacy of HAIC combined with PD-1 inhibitors plus lenvatinib in patients with advanced HCC. In the retrospective review, the median OS (15.9 vs. 8.6 months, respectively; HR 0.60, 95% CI, 0.43–0.83; *p* = 0.0015) and median PFS (8.8 vs. 5.4 months, respectively; HR 0.74, 95% CI, 0.55–0.98; *p* = 0.0320) were higher in the HAIC plus PD-1 inhibitors plus lenvatinib group compared with the PD-1 inhibitors plus lenvatinib group. Their conclusion demonstrated that the superiority of antitumour efficacy in the HAIC plus PD-1 inhibitors plus lenvatinib regimen over that in the PD-1 inhibitors plus lenvatinib regimen was significant. Although the mechanisms of response and resistance to the pembrolizumab plus lenvatinib regimen remain unclear [16], an explanation as to why a higher median OS (15.9 months) was detected may be that under the premise of pembrolizumab plus lenvatinib use, the earlier the HAIC is utilized, the greater the survival is detected for these patients with uHCC [18, 19]. Previous studies [23, 24] have shown that it is effective in early tumours, which may be attributed to the local tumour-killing effect of HAIC. However, for unresectable tumours, the role of HAIC is limited [9]. One hypothesis is that unresestable tumours have a mature mechanism to evade recognition by CD8+ T cells [16]. This mature mechanism is highly related to the immune escape of tumour cells [17]. This could explain why unresectable tumours are resistant to conventional chemotherapy. Those with early HCC undergoing HAIC therapy have a marked, durable response, implying some derive short-term benefit from HAIC [23].

Child-Pugh stage as a risk factor for survival may be associated with prognosis in patients with uHCC, implying that patients with poor liver function might fail to benefit from triple combination therapy given that survival is limited by liver function [11, 12]. Accordingly, hepatic lesion control is conducive to improving prognosis to a certain extent [11]. Although the efficacy of the pembrolizumab plus lenvatinib regimen in extending survival is encouraging [16], distinct differences have been detected in the duration of HAIC therapy [2]. Management of advanced uHCC by inhibiting or blocking the activation of tumour signal transduction pathways is a strategy to address malignant tumours, and it may represent a trend [8, 25, 26]. Pembrolizumab, a highly selective IgG4-kappa humanized monoclonal antibody targeting PD-1, is indicated for the management of patients with advanced microsatellite instability-high or mismatch repair deficiency, and PD-L1-positive expression is associated with improved survival [12, 17]. Lenvatinib inhibits the kinase activities of vascular endothelial growth factor receptors that are associated with pathogenic angiogenesis and tumour growth [16]. Lenvatinib indirectly decreases the number of total PD-1 receptors by reducing the number of cancer cells, reinforcing the effect of pembrolizumab by promoting cancer cells to enter into “a dormant state” [2, 15]. This synergistic effect of pembrolizumab and lenvatinib may represent the basis for HAIC’s effectiveness in managing advanced HCC.

uHCC frequently suffers from the overexpression of immune checkpoints, which is associated with tumour immune escape [3, 17]. Targeting this characteristic, pembrolizumab monotherapy has been shown to have a robust clinical benefit in patients with uHCC [12, 17]. However, no strategies to prevent immune checkpoint inhibitor resistance have been reported. Thus, systemic combination therapy in both first- and second-line settings for the management of patients with uHCC has become a recent strategy [3, 17, 27, 28]. Recently, atezolizumab (an anti-PD-L1 antibody) plus bevacizumab has been approved for the first-line management of uHCC in view of encouraging effectiveness from the IMbrave150 trial [27], which showed the estimated rate of survival at 12 months was 67.2% (95% CI, 61.3–73.1). Durvalumab is a humanized IgG antibody that binds PD-L1 and can block the interaction of PD-1 and PD-L1, eliminating inhibition of CD8+ T cell responses [29]. Durvalumab plus an anti-CTLA-4 antibody showed that the median OS reached 18 months in patients with uHCC [28]. A single-centre retrospective review [25] assessing ipilimumab plus anti-PD-1 inhibitor in patients with advanced HCC with progression on previous immune checkpoint inhibitors demonstrated that the median OS was 10.9 months (95% CI, 3.99–17.8), the median time-to-progression was 2.96 months (95% CI, 1.61–4.31), and the median duration of response was 11.5 (range, 2.76–30.3) months.

In the retrospective review, with patients who had treatment-naive uHCC exhibiting PD-L1 staining, no unpredicted AEs attributed to PLH or PL combination therapy were observed. Although the duration of PLH or PL combination therapy may be inconsistent in some patients, the inconsistent duration in these patients did not have contributed to the remarkable differences in the rates of key AEs. The rate of key AEs was similar to those seen in other studies [30, 31] of pembrolizumab combined with lenvatinib in patients with uHCC, implying that the toxicity profile of PLH or PL combination therapy tends to be controllable. AEs related to PLH or PL were in accordance with the well-known AEs reported in previous studies [2, 16, 32, 33].

There are several limitations to this final analysis. First, the retrospective nature of the present study has inherent biases. Survival results might have been affected by relatively small patient numbers, heterogeneous populations, selection biases, and subsequent management. The lack of revalidation of diagnostic procedures and a central blinded review may result in the overestimation of survival curves. Patients with depression, those who are potentially suicidal, or those performing acts of self-harm at home were not involved in the study. The final aetiological analysis of the deaths was not complete, which may lead to an underestimation of the survival curves. Second, mutational and other gene expression signatures and patient stratification based on primary HCC location were not performed in this study. Third, patients included in this study were limited to those patients with treatment-naive HCC exhibiting PD-L1 staining; hence, these results should not be generalized to all patients with HCC.

## Conclusion

The results described here may support the growing body of evidence showing that the incorporation of HAIC into pembrolizumab plus lenvatinib is associated with improved survival benefits in selected populations of patients with treatment-naive uHCC exhibiting PD-L1 staining. Nevertheless, because the present study was only a retrospective review, we could not arrive at any definitive conclusion regarding the utilization of pembrolizumab plus lenvatinib plus HAIC in the management of uHCC. A prospective multicentre study is being planned to validate these findings.

## Data Availability

The datasets used and/or analyzed during the current study fails to be publicly available because of privacy regulations but are available from the corresponding author on reasonable request.

## Abbreviations

HCC: hepatocellular carcinoma
HAIC: hepatic arterial infusion chemotherapy
PD-L1: programmed cell death ligand-1
PLH: pembrolizumab plus lenvatinib plus HAIC
PL: pembrolizumab plus lenvatinib
FOLFOX: oxaliplatin, fluorouracil, and leucovorin
OS: overall survival
PFS: progression-free survival
AEs: adverse events
HR: hazard ratio
CI: confidence interval
PD-1: programmed cell death protein-1
CTLA-4: cytotoxic T-lymphocyte-associated protein 4
BCLC: Barcelona Clinic Liver Cancer
CPS: combined positive score
ECOG: Eastern Collaborative Oncology Group
RECIST: Response Evaluation Criteria in Solid Tumors
ALBI: albumin–bilirubin
CT: computed tomography
MRI: magnetic resonance imaging
